# Efficient Video Compression Using Afterimage Representation

**DOI:** 10.3390/s24227398

**Published:** 2024-11-20

**Authors:** Minseong Jeon, Kyungjoo Cheoi

**Affiliations:** Department of Computer Science, Chungbuk National University, 1 Chungdae-ro, Seowon-gu, Cheongju 28644, Chungbuk, Republic of Korea; jeonminseong@chungbuk.ac.kr

**Keywords:** afterimage-based video compression, optical flow, keyframe selection, real-time video processing, resource-efficient computing, temporal context preservation

## Abstract

Recent advancements in large-scale video data have highlighted the growing need for efficient data compression techniques to enhance video processing performance. In this paper, we propose an afterimage-based video compression method that significantly reduces video data volume while maintaining analytical performance. The proposed approach utilizes optical flow to adaptively select the number of keyframes based on scene complexity, optimizing compression efficiency. Additionally, object movement masks extracted from keyframes are accumulated over time using alpha blending to generate the final afterimage. Experiments on the UCF-Crime dataset demonstrated that the proposed method achieved a 95.97% compression ratio. In binary classification experiments on normal/abnormal behaviors, the compressed videos maintained performance comparable to the original videos, while in multi-class classification, they outperformed the originals. Notably, classification experiments focused exclusively on abnormal behaviors exhibited a significant 4.25% improvement in performance. Moreover, further experiments showed that large language models (LLMs) can interpret the temporal context of original videos from single afterimages. These findings confirm that the proposed afterimage-based compression technique effectively preserves spatiotemporal information while significantly reducing data size.

## 1. Introduction

Recent advances in CCTV technology, driven by the adoption of high-resolution cameras, the integration of artificial intelligence (AI), and the deployment of cloud-based solutions, have led to significant improvements. The widespread availability of 4K-resolution cameras and the development of AI-powered object detection have substantially enhanced the accuracy and efficiency of surveillance systems. The adoption of cloud-based storage and analytics platforms has enabled real-time monitoring and remote access, while the advent of 5G networks has facilitated the seamless transmission of large-scale video data in real time [1]. As a result, CCTV systems have evolved into critical infrastructure across various sectors, including crime prevention and investigation, traffic management, disaster monitoring, and smart city development [2].

Despite these technological advancements and their diverse applications, existing surveillance systems continue to face significant challenges. A primary issue is the overwhelming increase in data processing and storage demands. The proliferation of high-resolution cameras has led to an exponential growth in the volume of video data generated. For instance, a single 4K-resolution camera can produce up to 70 GB of data per day, placing substantial strain on existing storage and processing systems. Furthermore, the real-time analysis of such large-scale video data remains a persistent challenge. Even with advancements in AI-driven object detection, processing vast amounts of video data requires considerable computational power, which remains a bottleneck in real-time surveillance.

One of the most pressing challenges for surveillance systems is detecting anomalous behaviors that occur sporadically over extended periods. This task demands continuous analysis of vast datasets while preserving temporal context. However, current systems are limited in their ability to efficiently compress and retain temporal information from long-term video streams without compromising analytical performance.

To address these challenges, there is a critical need for technologies that can efficiently compress and process large-scale video data, enhance real-time analysis capabilities, and summarize data while preserving temporal information. In this paper, we propose a video compression technique based on afterimage representation to effectively overcome these limitations.

**Figure 1:**
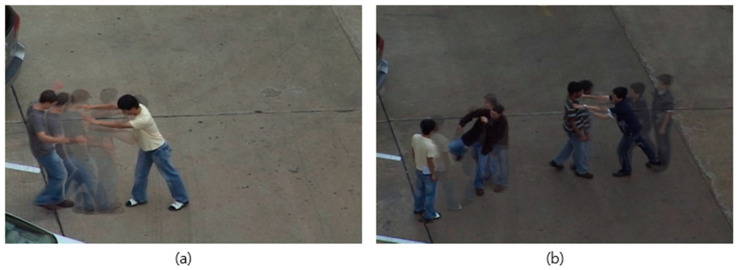
Example of afterimages: (**a**) an afterimage showing behavioral interactions within a single group; (**b**) an afterimage depicting simultaneous behavioral interactions between two groups.

Figure 1 illustrates examples of afterimages generated from the UT-Interaction dataset [3]. Figure 1a depicts an afterimage of a man in a yellow shirt pushing another person, while Figure 1b depicts an afterimage capturing the actions of two groups: one group on the right pushing an opponent, and another on the left kicking toward their opponent. As demonstrated in Figure 1, even a single afterimage provides a clear representation of the temporal motion dynamics that would otherwise require multiple video frames to visualize.

An afterimage is a technique that accumulates the movements of objects within a video over time to create a single image. This method efficiently reduces data size while preserving the critical visual information of the original video, enabling data compression without losing temporal context. An afterimage facilitates intuitive recognition of key motion changes without needing to replay the original video, offering significant advantages in handling and interpreting temporal contexts at the image level. Additionally, by minimizing redundant background information in each frame and focusing solely on changes between frames, it significantly enhances data storage and processing efficiency by reducing the overall data volume of the video.

Traditional video processing architectures, such as Transformer-based models [4] and 3D-CNNs [5], face substantial computational overhead due to the requirement to handle the temporal dimension. In contrast, the proposed afterimage generation technique reduces the complexity of video processing while effectively preserving temporal information. The core advantage of afterimages lies in their extreme data compression capabilities and the flexibility offered by their image format. For short videos, the entire sequence can be compressed into a single afterimage, enabling real-time processing using image-based models. For longer videos, a sequence of representative afterimages can serve as input to video processing models. This approach retains essential temporal information while maximizing computational efficiency, meeting a variety of video processing requirements.

Moreover, this advantage is crucial in overcoming the limitations of large multi-modal models (LLMs). Currently, models like GPT-4 [6] and Claude [7] are designed to handle image data but lack the capability to directly process video data due to the high computational costs involved in learning and analyzing temporal dynamics in videos. The use of afterimage techniques offers a potential solution by enabling interaction between LLMs and video data.

In this paper, we introduce a novel afterimage-based video compression technique that effectively compresses temporal information into a single frame, significantly reducing data volume. Our approach adaptively selects keyframes based on scene complexity using optical flow analysis, capturing dynamic actions with minimal redundancy. By generating object masks that isolate and accumulate moving objects over time, the resulting afterimages preserve essential spatiotemporal information. Experiments on the UCF-Crime dataset demonstrate that our method achieves an impressive average compression rate of 95.97%, reducing data size to roughly 1/25 of the original without compromising analytical performance. The compressed videos match the original in binary classification accuracy and outperform them in multi-class classification, particularly for detecting abnormal behaviors. Additionally, we show that large language models (LLMs) can interpret temporal context from single afterimages, suggesting promising applications in semantic video analysis and natural language understanding. Our approach addresses major challenges in managing large-scale video data, enhancing compatibility with AI models, and presenting impactful implications for real-time and resource-efficient computing in surveillance and related domains.

The structure of this paper is organized as follows: Section 2 reviews the related research, providing context and background for the study. Section 3 offers a detailed explanation of the afterimage generation process. Section 4 presents the experimental results, demonstrating the effectiveness of the proposed approach. Section 5 summarizes the overall findings and discusses potential future research directions.

## 2. Related Work

In this section, we review techniques that leverage afterimages by analyzing the composition and characteristics of video datasets used for action recognition, examining existing studies that have utilized afterimages, and exploring previous research on keyframe selection, which is crucial for generating afterimages. Additionally, we highlight the necessity of the proposed method based on prior studies and underscore its distinctiveness compared to previous approaches.

Video datasets are typically classified into trimmed and untrimmed videos, each requiring different analytical strategies. Trimmed videos consist of keyframes that correspond to a specific action or event, making them directly applicable to action recognition or video classification tasks. Common datasets in this category include UCF-101 [8], HMDB51 [9], and ActivityNet [10], which are characterized by minimal extraneous information, allowing them to be used for model training without extensive preprocessing. As a result, trimmed video datasets are frequently employed in action recognition studies that utilize models such as C3D [11], I3D [12], and Two-Stream CNN [13]. In contrast, untrimmed video datasets contain non-continuous actions throughout the video and often include segments irrelevant to action recognition, such as background scenes or unrelated objects like people, vehicles, or animals moving without meaningful interaction. Therefore, action recognition on untrimmed videos requires additional preprocessing, including temporal action localization. Notable datasets in this category include UCF-Crime [14] and Charades [15].

Afterimage techniques are particularly effective in handling the complexity of untrimmed videos. By highly compressing temporal information across multiple frames and eliminating unnecessary segments, afterimages enable the effective visualization of key actions. In datasets like UCF-Crime, where the environment remains static, the impact of afterimage techniques is maximized, as the consistent background facilitates a clearer representation of object movements.

The concept of afterimages is grounded in visual persistence, where the continuous positions of moving objects are overlaid into a single image. Ardianto et al. [16] employed frame differences to create motion trails in traffic monitoring videos, applying afterimage techniques to vehicle detection and tracking. This approach achieved comparable accuracy to video object detectors while tripling processing speed and reducing model size by 28%. However, since this method used all frames to generate afterimages without selectively choosing keyframes, its data compression efficiency was limited. A study by Eschner et al. [17] introduced an Illustrative Motion Smoothing technique, which applies geometric smoothing and motion blur to complex 3D visualizations. This method focuses visual attention on key objects by smoothing the motion of background elements, emphasizing temporal information, and removing unnecessary data, similar to the principles underlying afterimage techniques.

While afterimage techniques show significant potential in compressing temporal information in videos, research that specifically implements this approach in video compression or analysis remains limited. In particular, studies on the mechanisms for selecting important frames, which are crucial for afterimage generation, are sparse. Research on frame selection in video summarization provides a valuable reference for the process of generating afterimages. Nahian et al. [18] proposed a CNN-based model that learns the visual features of each frame to predict its importance, thereby improving the accuracy of keyframe selection. Almeida et al. [19] introduced a video summarization method based on the compressed domain, using DC images and HSV color histograms extracted from I-frames of MPEG videos to calculate frame similarity, enabling real-time processing without fully decoding the video. Similarly, the SUM-GAN-AAE model proposed by Apostolidis et al. [20] enhanced keyframe selection through attention mechanisms, learning the relationships between frames to more effectively identify keyframes. SCSampler [21] proposed a sampling technique that reduces computational costs by selecting key clips in a video, using a CNN-based importance prediction module to estimate how much each clip contributes to action recognition. AdaFrame [22] introduced a method that updates the current state using a global context comprising the current frame, previous states, and spatiotemporal information, enabling adaptive frame selection while maintaining high accuracy. Lastly, Mamba [23] developed a Selective State Space Model (SSM) that can focus on or ignore specific information based on input, efficiently modeling sequence data.

While previous studies on keyframe selection and video summarization offer valuable insights, they do not fully meet the specific requirements of afterimage generation. Traditional methods focus on individually significant frames or major scene changes to summarize content effectively. In contrast, afterimage generation necessitates selecting keyframes that capture motion continuity and preserve temporal dynamics cohesively. This process must consider the flow of motion between frames to ensure the resulting afterimage accurately reflects the movement patterns and temporal structures of the original video.

Recent advancements in artificial intelligence have seen large language models (LLMs) like GPT-4 and Claude begin to tackle video-related tasks, while diffusion models have achieved unprecedented progress in video generation. The introduction of Denoising Diffusion Probabilistic Models (DDPMs) by Ho et al. [24] established fundamental principles that have been rapidly expanded for video applications [25,26,27,28,29,30], marking significant advancements in this field. Despite these rapid strides in video-related research, the core technology remains heavily grounded in image-processing techniques. Due to the complexity and high computational demands of video data, even large-scale models often rely on image-based approaches to remain feasible, underscoring the ongoing challenge of efficient video processing in AI systems.

Afterimages offer a unique advantage in addressing these computational challenges by inherently compressing temporal information into a single frame. For large-scale models like LLMs and diffusion models that predominantly operate on image-based inputs, afterimages provide an efficient method for processing video content while preserving the computational efficiency of image-based processing. Their ability to capture motion continuity and temporal structures in a single frame allows for diverse video applications without necessitating the processing of entire video sequences. This approach is particularly beneficial in tasks such as video restoration, where maintaining accurate temporal dynamics is essential, and in video generation, where temporal consistency is critical. By leveraging afterimages, these models can efficiently manage video-related tasks while operating within the limitations of image-based architectures, offering a promising solution to the computational complexities inherent in video processing for AI systems.

In this paper, we propose an optical flow-based motion analysis and an adaptive keyframe selection mechanism to meet these requirements. In segments with significant movement changes, more keyframes are selected to capture the continuity of the motion in detail, while in segments with minimal movement changes, fewer keyframes are chosen to maintain the overall flow of behavior. This approach preserves more information during dynamic actions and compresses data more effectively when changes are minimal. Consequently, the proposed method optimizes keyframe selection for afterimage generation, enabling the efficient encoding of critical temporal information from videos into afterimages.

## 3. Methodology

This section provides a detailed explanation of the proposed afterimage generation method. The method aims to effectively summarize the temporal information of a video and compress it into an afterimage by undergoing a series of processes to select important scenes based on the analysis of object movement from the input video and visually integrating these selected scenes.

**Figure 2:**
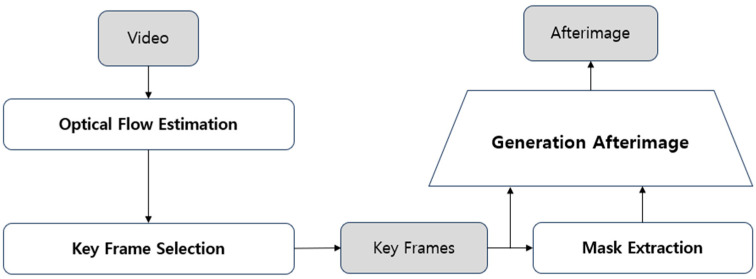
Workflow of the proposed afterimage generation pipeline.

Figure 2 illustrates the workflow of the proposed afterimage generation pipeline, which consists of four steps. In the first step, optical flow is calculated to analyze the motion between successive frames of the input video, generating pixel-level motion vectors that capture the dynamic characteristics of the objects within the scene. In the second step, an adaptive keyframe selection module identifies keyframes using the previously calculated optical flow information. Keyframes are selected at points where significant changes in motion or transitions in action patterns occur. The number of keyframes is adaptively determined based on the complexity of the scene—more keyframes are selected for scenes with complex or significant action changes, while fewer keyframes are chosen for simpler scenes with minimal changes, thereby achieving efficient data compression. In the third step, object masks are generated by identifying moving objects in the selected keyframes and separating them from the background. These masks enhance the quality of the afterimage by clearly representing the motion of the objects. In the fourth step, the generated object masks are accumulated over time, and alpha blending is applied to produce the final afterimage. This process effectively visualizes the changes in object motion at each time step while minimizing redundancy, resulting in a clear representation of the object’s movement trajectory.

In the following sections, each module of the proposed method is thoroughly explained with corresponding mathematical formulations. At the end of each section, the procedures are summarized in pseudocode, presented as Algorithms 1–4.

### 3.1. Optical Flow Estimation

The first step in afterimage generation involves calculating optical flow to analyze the dynamic characteristics of the video sequence. Optical flow is a widely used technique in computer vision for tracking object movement between consecutive frames by computing the motion vectors of pixels based on brightness changes. This technique enables a quantitative analysis of object movement within the video.

Various algorithms [31,32,33] have been developed for optical flow calculation in the field of computer vision. In this study, we utilize the Farnebäck algorithm [33] to estimate the dynamic information in the video. The Farnebäck algorithm is a dense optical flow estimation method that uses polynomial expansion to approximate motion between frames. It supports multi-scale analysis, allowing it to handle large-scale motion across a broad range. By balancing speed and accuracy, this algorithm is well suited for real-time applications, particularly when processing large-scale images with complex motion.
**Algorithm 1: Optical Flow Estimation**
**Input**: Video sequence $V={\{I}_{1},I_{2},\ldots ,I_{N}\}$ **Output**: Magnitude of optical flow $M=\{M_{1},M_{2},\ldots ,M_{N-1}\}\;$1**Begin**2For t=1 to N−1 **do**3   Compute the Magnitude of optical flow between frames:4   $M_{t}=||OF(I_{T},\;I_{T+1})||$
5**End**

### 3.2. Adaptive Keyframe Sampling

In the second step, keyframes are selected based on the previously calculated optical flow. The proposed keyframe selection algorithm precisely analyzes the dynamic characteristics of motion in the video to identify significant temporal changes. To achieve this, it utilizes an EWMA (Exponentially Weighted Moving Average) and a Gaussian filter. Additionally, a novel method incorporating periodic variability is introduced to prevent the loss of temporal information in static segments. This keyframe selection algorithm is specifically designed to effectively capture dynamic temporal changes in the video, as described in Equation (1).

In Equation (1), C(t) represents a composite function that describes the temporal order of video frames. It is defined as the product of time t and the absolute value of the second derivative of the sum of G(t) and S(t).

$$C\left(t\right)=t\cdot \left.\left|\frac{d^{2}}{dt^{2}}\left[G\left(t\right)+S\left(t\right)\right]\right.\right|$$

Here,

(1) $$\;G(t)=Gaussian(EWMA\left(M\left(t\right),\sigma \right),M\left(t\right)=||OF(I\left(t\right),I\left(t+1\right))||,$$

$$S\left(t\right)=A\cdot Sin\left(\omega \cdot t\right),\;\omega =\frac{2\pi }{T}$$

G(t) quantifies the intensity of temporal changes in the video, calculated using optical flow between consecutive frames. The value of G(t) varies with scene complexity and the magnitude of motion, and it is used to detect significant temporal changes in the video. Specifically, G(t) is obtained by applying a Gaussian filter to the Exponentially Weighted Moving Average (EWMA) of the optical flow magnitude M(t), where $M\left(t\right)=||OF\left(I\left(t\right)+I\left(t+1\right)\right)||$. Here, *OF*(*I*(*t*)*, I*(*t* + 1)) is the function that calculates the optical flow between two consecutive frames, *I*(*t*) and *I*(*t* + 1), and ||·|| denotes the magnitude of the calculated flow. Applying the *EWMA* to this magnitude enables tracking of the trend in motion changes over time. The *EWMA* assigns a higher weight to recent movements while still reflecting past movement information, allowing the algorithm to account for both short-term patterns of abrupt movements and long-term patterns of gradual motion changes. To generate a smooth curve by mitigating sharp changes or noise, a Gaussian filter, Gaussian (·,σ), is applied to the *EWMA* values, resulting in *G*(*t*). Here, σ is the standard deviation of the Gaussian filter, which controls the intensity of noise reduction. This process transforms discrete optical flow information into a differentiable continuous function. The term *S*(*t*) represents a sine function that introduces periodic variability into the system, where A denotes the amplitude and ω represents the angular frequency defined as $\omega =\frac{2\pi }{T}$, with T representing the period. In this study, ω is set to 0.1. By incorporating a sine function into *G*(*t*), the method maintains temporal consistency, captures periodic actions, and ensures balanced sampling. This approach is particularly beneficial for representing static scenes or repetitive motion patterns over extended periods. Finally, C(t) is computed by multiplying time t with the absolute value of the second derivative of the sum of *G*(*t*) and *S*(*t*), as shown in Equation (1), which characterizes the temporal order of video frames.

**Figure 3:**
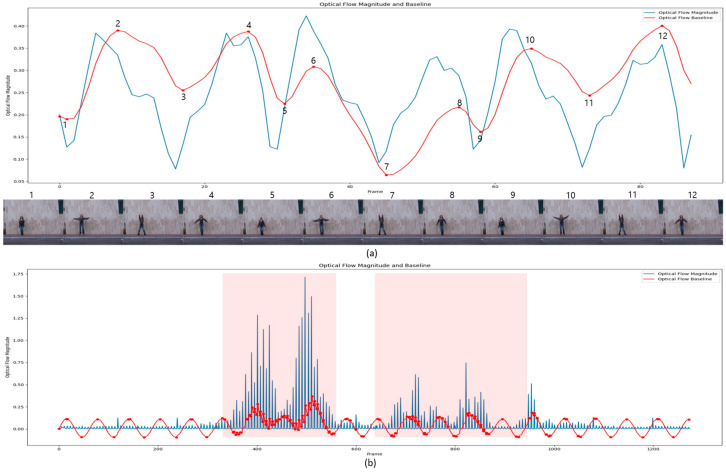
Results of applying the proposed keyframe selection algorithm: (**a**) keyframe extraction from the ‘daria_jack’ video in the Weizmann dataset; (**b**) keyframe extraction from the ‘Burglary034_x364’ video in the UCF-Crime dataset.

The proposed method’s key strength lies in its ability to transform non-linear time–motion relationships into an analyzable function. This transformation facilitates the dynamic selection of more keyframes in areas with significant motion changes and fewer keyframes in more static regions. As a result, the method efficiently minimizes redundancy while accurately capturing critical temporal variations. These characteristics are illustrated in detail in Figure 3.

Figure 3 illustrates the working principle and effectiveness of the keyframe selection algorithm. In the graph, the blue line represents the magnitude of the optical flow, while the red line depicts the composite function *C*(*t*), calculated according to Equation (1). The circled points with numbers indicate the keyframes selected by the algorithm.

Figure 3a shows the keyframe extraction results for the ‘daria_jack’ video from the Weizmann dataset [34]. This video is about 3 s long (approximately 100 frames) and features a woman performing jumping jacks. The sequence of images at the bottom displays the 12 keyframes selected by the proposed algorithm, which accurately capture the main stages of the jumping jack motion: (1) the starting phase, where the arms are lifted, (2) the peak moment, where the arms are fully raised, and (3) the ending phase, where the arms are lowered. As shown in the graph in Figure 3a, the peaks in optical flow magnitude and the inflection points in *C*(*t*) align precisely with the selected keyframes. This result indicates that the proposed method effectively captures key motion changes in the action sequence.

Figure 3b displays the keyframe extraction results for the ‘Burglary034_x364’ video (approximately 1300 frames) from the UCF-Crime dataset. The results demonstrate how the proposed algorithm adaptively adjusts the selection of keyframes based on the varying intensity of motion. The video depicts a burglary incident at a store, followed by a chase between the store owner and the burglar. The two pink-highlighted areas in Figure 3b represent the frames where the burglary occurs (frames 350–550) and the subsequent chase (frames 650–900). In these regions, *G*(*t*) rises sharply, prompting the algorithm to select more keyframes to capture the intense motion during these critical events. In contrast, the other regions contain static segments with minimal motion. In these areas, the influence of *G*(*t*) decreases, and the periodic variability from *S*(*t*) becomes more prominent, resulting in the selection of one keyframe approximately every 25 frames. This approach ensures that even in static segments, the algorithm maintains temporal continuity by sampling frames at regular intervals due to *S*(*t*). The effectiveness of the proposed adaptive keyframe selection algorithm is demonstrated in Figure 3. This algorithm consistently adapts across varying time scales. In the short term, it identifies inflection points within action patterns, selecting keyframes at moments of significant motion changes, thereby enabling the efficient extraction of critical transitions in each action. When applied to longer time frames, the algorithm tends to select more keyframes in segments with frequent events while maintaining regular intervals in static regions, based on the frequency of keyframes detected within the action patterns.
**Algorithm 2: Adaptive Keyframe Sampling**
**Input**: Magnitude of optical flow $M=\{M_{1},M_{2},\ldots ,M_{N-1}\}\;$ **Output**: Keyframe indices
K1**Begin**2 Compute the EWMA of M: ${EWMA}_{M}=EWMA(M,\alpha )$
3 Apply Gaussian smoothing to obtain $G_{t}$
$:\;G_{t}=Gaussian({EWMA}_{M},\sigma )$
4 Compute the periodic component $S_{t}$
$:\;S_{t}=A\cdot \sin\left(\omega \cdot t\right),\;\;\omega =\frac{2\pi }{T}$
5 Compute the composite function $C_{t}$
$:\;C_{t}=t\cdot \left.\left|\frac{d^{2}}{dt^{2}}\left[G_{t}+S_{t}\right]\right.\right|$
6 Identify keyframes at local maxima of $K=\{t|C_{t}\;is\;a\;local\;maximum\}$
7**End**

### 3.3. Object Mask Generation

In this stage, object masks are generated based on the extracted keyframes to create effective afterimages. Object masks are designed to eliminate redundant background information from the keyframes, isolating object motion for clearer tracking and representation. The process involves performing object segmentation on the selected keyframes, extracting the regions corresponding to the objects, and generating binary masks that accurately represent the shape and position of the objects in each keyframe. By excluding unnecessary background details, this approach preserves only the essential moving object data. The detailed process for generating object masks is outlined in Equation (2).

(2) $$M\left(t,x,y\right)=\left\{\begin{array}{c} \;1,\;\;\;\;\;if\;\left|OF\left(t,x,y\right)\right|>\tau \\ 0,\;\;\;\;\;otherwise\;\;\;\;\;\;\; \end{array}\right.$$

In Equation (2), $M\left(t,x,y\right)$ represents the object mask at time t, while OF(t,x,y) denotes the magnitude of the motion vector obtained from the optical flow. The threshold τ used in this study is set to 0.2. This thresholding process filters out minor movements or noise, resulting in a mask that includes only the regions where objects exhibit movement exceeding the threshold. Consequently, the generated mask minimizes unnecessary background information and enhances the clarity of object motion, thereby improving the quality of the afterimage.
**Algorithm 3: Object Mask Generation**
**Input**: Keyframes
$\left\{I_{k}|k\in K\right\}$, Optical flow ${OF}_{k}|k\in K\}$, Threshold τ **Output**: Object masks
$\left\{M_{k}|k\in K\right\}$1**Begin**2**For each** keyframe $I_{K}:$
3 **For each** pixel $\left(x,y\right):$
4 Compute: $M_{k}\left(x,y\right)=\left\{\begin{array}{c} 1,\;\;if\left|\left|{OF}_{k}\left(x,y\right)\right|\right|>\tau \\ 0,\;\;otherwise\;\;\;\;\;\;\;\;\;\;\;\;\;\;\;\;\; \end{array}\right.$
5**End**

### 3.4. Temporal Mask Synthesis for Afterimage Generation

The final step in afterimage generation involves accumulating the object masks from each keyframe in temporal order to create the complete afterimage. During this process, overlapping regions from each time step are removed, and the remaining unique changes in object movement are blended using an alpha blending technique. This approach ensures that the movement trajectory of the object over time is clearly depicted by highlighting only the changes in the object that do not overlap with the previous frame. The following equations (Equations (3)–(5)) describe the entire process.

In Equation (3), M(t,x,y) is the object mask at time t, and M′(t,x,y) denotes the object mask with overlapping areas removed.

(3) M′(t,x,y)=M(t,x,y)·(1−M(t−1,x,y))

The right-hand side of Equation (3) calculates the negation of the previous frame mask at t−1 and multiplies it by the current mask, ensuring that only new, previously unrepresented areas are included. This approach highlights the changes in object movement more clearly and prevents overlapping information in the same location.

Equation (4) computes the alpha blending coefficient, denoted as $\alpha_{t}$.

(4) $$\alpha_{t}=1-\gamma (n-t)$$

Here, $\alpha_{t}$ is the alpha blending coefficient for frame t, and n represents the number of keyframes used to create a single afterimage. In our experiment, seven keyframes were generated as one afterimage. The parameter γ is a hyperparameter, set to 0.15 in our experiment. Through Equation (4), $\alpha_{t}$ becomes 1 in the most recent frame and decreases by a factor of γ as it moves to earlier frames. The alpha blending coefficient controls the transparency of the object motion over time, ensuring that more recent movements are more prominently displayed.

Equation (5) describes the process for generating the final afterimage.

(5) $$I_{afterimage}(x,y)=O(n,x,y)+\sum_{t=1}^{n-1}\alpha_{n-t}\cdot M\prime (n-t,x,y)\cdot O(n-t,x,y)$$

Here, $I_{afterimage}$ represents the final afterimage, while O(n,x,y) denotes the original image of the most recent keyframe. Equation (5) accumulates the original information from O(n−1,x,y), aligning the regions of the object mask M′(n−t,x,y) with the alpha coefficient $\alpha_{n-t}$ over time. The result is a final afterimage that visualizes the trajectory of the object’s motion, while redundant background information is removed, leaving only the most important object movements in temporal order.

The computational complexity of the proposed afterimage generation method is O(N·H·W), primarily driven to the optical flow calculations, where N denotes the total number of frames, and H and W represent the height and width of each frame, respectively.

**Figure 4:**
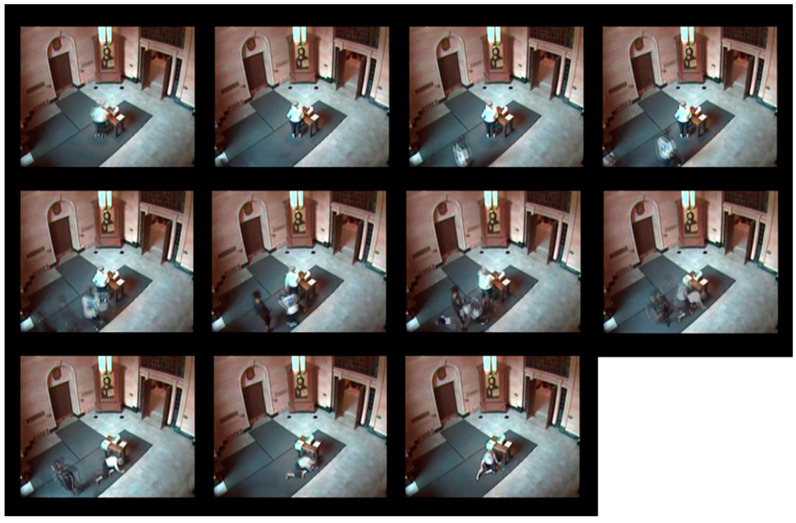
Chronological sequence of afterimages generated from the ‘Abuse001_x264’ video. **Top row**: afterimages 1–4; **middle row**: afterimages 5–8; **bottom row**: afterimages 9–11.

Figure 4 presents the afterimages generated from the first video in the UCF-Crime dataset, titled ‘Abuse001_x264’. This video consists of 761 frames, from which the proposed algorithm extracted 75 keyframes, resulting in the generation of 11 afterimages. Figure 4 displays 11 selected afterimages. Each afterimage overlays multiple keyframe data points to effectively depict changes in the scene over time within a single image.
**Algorithm 4: Afterimage Generation**
**Input**: Keyframes $\left\{I_{k}|k\in K\right\}$, Object masks $\left\{M_{k}|k\in K\right\}$, Blending coefficients $\left\{a_{k}\right\}$ **Output**: Afterimage $I_{afterimage}$1**Begin**2 Initialize the afterimage with the last keyframe: $I_{afterimage}=I_{last\_keyframe}$
3 **For**
$k=last_{keyframe}-1$ **down to** first:4   Compute the non-overlapping mask: ${M\prime }_{k}=M_{k}\cdot (1-M_{k+1})$
5   Update the afterimage: $I_{afterimage}=I_{afterimage}+a_{k}\cdot {M\prime }_{k}\cdot I_{k}$
6**End**

## 4. Experiments

### 4.1. Datasets

To evaluate the performance of the proposed system, experiments were conducted using the UCF-Crime dataset. The UCF-Crime dataset is a large-scale anomaly detection dataset derived from real surveillance camera footage. It consists of thirteen classes of abnormal behaviors (Abuse, Arrest, Arson, Assault, Burglary, Explosion, Fighting, Road Accidents, Robbery, Shooting, Shoplifting, Stealing, and Vandalism) and one class representing normal behavior. The dataset contains 1900 videos, with durations ranging from a few seconds to several minutes.

The primary reason for selecting the UCF-Crime dataset is its inclusion of untrimmed videos, which closely resemble real-world surveillance footage. This dataset features a mix of various actions, as well as scenes with only background activity or no significant actions, mirroring the characteristics of long, unedited video data typically found in surveillance environments. Additionally, most videos are recorded using fixed cameras, making the dataset ideal for evaluating the proposed afterimage generation method.

For dataset partitioning, the videos were split into training, validation, and test sets in a ratio of 8:1:1, ensuring a balanced distribution across all classes. This approach mitigates issues related to the relatively small number of videos in certain classes, which could otherwise impact the reliability of performance evaluations for those specific categories. Specifically, of the 1900 total videos, 1520 were allocated to the training set, 190 to the validation set, and the remaining 190 to the test set.

The experiments were conducted on an NVIDIA A100 GPU with 80 GB of memory. A batch size of 16 was selected to optimize GPU’s memory usage.

### 4.2. Experiments on and Results of Generating Compressed Video Based on Afterimage

An experiment was conducted to compare the file size of the original videos with the compressed videos generated using the proposed afterimage method on the UCF-Crime dataset. Table 1 presents a comparison between the compression rates of videos consisting only of keyframes and those consisting solely of the final afterimages for each class.

As shown in Table 1, the proposed afterimage-based compression method achieved a significantly higher compression rate compared to the original videos. Among the anomaly classes, ‘Burglary’ exhibited the highest compression rate, with 84.60% compression at the keyframe stage and 96.92% at the afterimage stage. In contrast, ‘Fighting’ had the lowest compression rate, achieving 70.21% at the keyframe stage and 94.04% at the afterimage stage. This lower rate is due to the abrupt changes in movement during fighting scenes, which require the selection of more keyframes to preserve critical information. Overall, the keyframe extraction phase alone resulted in an average compression rate of 79.83%, while the afterimage generation phase produced a final compression rate of 95.97%. This indicates that the proposed afterimage-based compression method can reduce the size of videos to approximately 1/25 of their original size on average for the UCF-Crime dataset.

### 4.3. Experiments and Results for Quantitative Evaluation

The high compression rate observed in Section 4.2 indicates that the proposed method offers a novel approach to saving significant storage space and processing time in large-scale surveillance systems. However, such extreme compression is only valuable if the key information from the video is preserved. Therefore, an additional experiment was conducted to assess whether the proposed afterimage-based compression method retains the essential information from the original video.

To evaluate the balance between data efficiency and classification accuracy, binary and multi-class classification experiments were performed using both the original videos and the compressed videos generated with afterimages. The SlowFast [35] model was employed for these classification experiments, chosen for its capability to process video frames directly as input without requiring additional feature extraction. This approach minimizes performance variability due to external factors, allowing for a pure comparison between the original and afterimage-compressed videos. Consistent hyperparameters and training settings were applied to both video types to ensure a fair comparison.

Table 2, Table 3 and Table 4 present the results of binary and multi-class classification experiments using the UCF-Crime dataset, comparing original videos with afterimage-based compressed videos. Before analyzing the results, it is important to note that the UCF-Crime dataset exhibits significant class imbalance, with the ‘normal’ class being considerably more prevalent than the ‘abnormal’ classes. Additionally, ‘normal’ class videos are characterized by longer sequences and less movement compared to videos depicting anomalous behaviors.

Table 2 presents the results of binary classification experiments. The proposed afterimage-based videos demonstrated performance comparable to, though slightly lower than, the original videos. This can be explained as follows: In the classification process using original videos, the method involves dividing the videos into clips and sampling frames at regular intervals. Given the characteristics of the UCF-Crime dataset, where ‘normal’ class videos predominantly consist of sequences with minimal or no action, this sampling method often selects frames with little to no movement. In contrast, the proposed afterimage-based compression method focuses on selecting keyframes with significant motion, resulting in afterimages that primarily capture movement information. As a result, the original video model tends to learn based on the presence or absence of motion, while the afterimage-based model learns from the characteristics of the motion itself, which aligns more closely with the purpose of anomaly detection. Therefore, in the binary classification task of distinguishing between ‘normal’ and ‘abnormal’, the lack of motion in the original videos may positively influence the classification of ‘normal’ instances, resulting in slightly higher performance. However, for the more complex task of distinguishing specific anomalous behaviors in multi-class classification, the afterimage-based method proves more effective. The results of these multi-class classification experiments are presented in Table 3 and Table 4.

Table 3 presents the results of multi-class classification experiments involving thirteen classes of abnormal behaviors and one class representing normal behavior. While binary classification simply distinguishes between normal and abnormal, multi-class classification requires distinguishing between normal behavior and 13 various types of abnormal behaviors. The results in Table 3 indicate that afterimage-based compressed videos outperform original videos across all evaluation metrics. Table 4 presents the results of multi-class classification focused solely on the ‘abnormal’ classes, excluding the ‘normal’ class. This experiment was designed to eliminate bias caused by class imbalance, enabling a more accurate evaluation of classification performance among abnormal behaviors. The results show that afterimage-based compressed videos achieved a significant 4.25% improvement in the classification of 13 different types of abnormal behaviors compared to the original videos.

These results demonstrate that the afterimage-based compression method is effective in capturing subtle pattern differences among various types of behaviors and serves as a representation format capable of efficiently encoding temporal information. Consequently, the proposed method can effectively summarize critical information within complex video sequences, providing practical benefits for tasks such as diverse action recognition and anomaly detection.

### 4.4. Experiments and Results for Qualitative Evaluation

An experiment was conducted to evaluate the practicality and interpretability of the proposed afterimage technique. In this experiment, GPT-4 was provided with afterimages and prompts as inputs, without any additional training, and asked to generate detailed descriptions of the images. The experiment utilized datasets with varying complexities and characteristics. Specifically, the Weizmann dataset, UCF-Crime dataset, and selected videos recording actual accidents were used. The use of these diverse sources was intended to encompass a wide range of scenarios, from simple to complex backgrounds, and from simple to complex actions. Each video was transformed into an afterimage using the proposed method, aiming to assess the applicability and effectiveness of the afterimage technique across various environments and behavior patterns. The primary goals of this experiment were twofold: first, to determine whether afterimages can effectively compress and represent the temporal and spatial information of a video, and second, to qualitatively assess whether a large language model (LLM) like GPT-4 can accurately interpret these afterimage representations and extract temporal context without any additional training.

Table 5 shows the results of a prompt-tuned GPT-4 model analyzing the content of afterimages created using the proposed afterimage-based video compression technique.

The afterimage in Table 5a was generated from a video depicting a man bending down and then straightening up on a street. When this afterimage was presented, GPT-4 accurately identified the sequence of movements with phrases like ‘stopped’, ‘bent down’, and ‘interacted with the ground’, recognizing that the individual paused, bent over, and then stood back up. These results indicate that the afterimage effectively compressed and preserved these simple movements in a temporal sequence, enabling GPT-4 to interpret the actions accurately.

The afterimage in Table 5b was generated from a video showing two men fighting in front of stairs on the right side, while a woman moves away on the left. Upon analyzing this afterimage, GPT-4 described the scene with expressions such as ‘aggressive or forceful movements’ and ‘individuals appear to be pushing or shoving each other within a confined space’, demonstrating its accurate understanding of the interaction among multiple individuals. These findings suggest that the afterimage successfully captured key interactions in the complex scene, enabling GPT-4 to interpret the sequence accurately.

The afterimage in Table 5c was generated from a video in which a man is lying on the ground while another man assaults him. Upon analyzing this afterimage, GPT-4 used descriptions like ‘vigorous physical action, such as a struggle or fight’ clearly recognizing the physical interaction between the two individuals. These results indicate that the afterimage effectively visualized the complex physical interaction, enabling GPT-4 to describe the event accurately.

Overall, the results in Table 5 demonstrate that the proposed afterimage-based video compression technique effectively compresses and represents both the temporal and spatial information of a video. This approach shows promise for applications in safety monitoring and accident analysis within complex industrial and surveillance environments. Moreover, the ability of LLMs like GPT-4 to accurately interpret the compressed representation in afterimages underscores the potential of using afterimages as a novel tool for video analysis in resource-constrained settings. This significant finding highlights the potential of afterimages as a new visual representation method that transcends simple data compression by spatially encoding the temporal information of video.

### 4.5. Significance and Implications of the Experimental Results

The series of experiments conducted in this study validated the effectiveness and potential of the proposed afterimage-based video compression technique from multiple perspectives. The findings offer the following key insights and implications.

First, the technique achieved a high compression rate while preserving essential information from the videos. Experimental results on the UCF-Crime dataset revealed that afterimage-based compressed videos reached an average compression rate of 95.97%, reducing the size of the original video data to approximately 1/25 of its initial size.

Second, the results of binary and multi-class classification experiments clearly demonstrate the effectiveness and potential of afterimage-based compressed videos. While maintaining performance comparable to the original videos in binary classification, they achieved superior results in multi-class classification. The 4.25% improvement in classifying abnormal behaviors, excluding the ‘normal’ class, is particularly noteworthy. This suggests that the proposed method can effectively capture and represent complex behavioral patterns and subtle differences, indicating that, despite the extremely high compression rate, key information from the videos is successfully preserved. As a result, this technology offers practical advantages for large-scale surveillance systems, including storage space savings, reduced network bandwidth usage, and enhanced real-time processing capabilities.

Third, the qualitative evaluation using large language models (LLMs) demonstrated that afterimages effectively encode the temporal and spatial information of a video. The ability of LLMs to accurately interpret complex action sequences, multiple object interactions, and the overall context of a scene from afterimages suggests that this technique opens new possibilities for semantic understanding and interpretation of video content. The integration of afterimages with LLMs could enable advanced natural language-based analysis of video data, paving the way for more intelligent and efficient video analysis systems.

## 5. Conclusions

This paper introduces an afterimage-based video compression technique that efficiently compresses the spatiotemporal data in videos. This method presents a novel solution to the challenges of data storage, processing, and analysis in large-scale surveillance systems. The primary advantage of afterimages lies in their ability to effectively encode a video’s temporal information into a single frame, resulting in enhanced data compression, real-time processing, and long-term behavior pattern analysis. Furthermore, the integration with large language models (LLMs) enables advanced natural language-based analysis and interpretation of video content, significantly enhancing the intelligence of video analysis systems.

However, the proposed technique has some notable limitations. The most significant constraint is its dependency on environments where the background remains relatively static or changes only minimally. While this makes the technique particularly effective for fixed-camera scenarios like CCTV, it poses challenges when applied to general videos with dynamic backgrounds. Additionally, a standardized definition of what constitutes a ‘good afterimage’ is still lacking. Comprehensive research is required to evaluate afterimage quality, taking into account factors such as the preservation of temporal information, visual clarity, and the ability to represent the essential content of the original video.

To further advance this technique, future research should focus on developing dynamic afterimage generation methods leveraging deep learning models. Integrating deep learning-based optical flow estimation could significantly improve computational efficiency by reducing the complexity associated with traditional approaches.

## Figures and Tables

**Table 1:** Class-specific compression rates of the proposed method for the UCF-Crime dataset.

Class	Keyframe Compression	Afterimage Compression	Class	Keyframe Compression	Afterimage Compression
Abuse	82.53%	96.51%	Robbery	78.61%	95.72%
Assault	72.11%	94.42%	Road Accidents	79.89%	95.98%
Arrest	76.24%	95.25%	Shooting	82.34%	96.47%
Arson	81.07%	96.21%	Vandalism	83.06%	96.61%
Burglary	84.60%	96.92%	Stealing	83.12%	96.62%
Fighting	70.21%	94.04%	Shoplifting	80.56%	96.11%
Explosion	78.93%	95.79%	Normal	84.41%	96.88%

**Table 2:** Binary classification experiment results (normal/abnormal).

Metric	Original Video	Afterimage-Based Video	Difference
Precision	0.7721	0.7618	−0.0103
Recall	0.7596	0.7411	−0.0185
F1-score	0.7641	0.7471	−0.0170
Accuracy	77.85%	76.58%	−1.27%

**Table 3:** Multi-class classification experiment results (1): fourteen classes (thirteen classes of abnormal behaviors and one class representing normal behavior).

Metric	Original Video	Afterimage-Based Video	Difference
Precision	0.1731	0.1955	+0.0224
Recall	0.1769	0.1874	+0.0105
F1-score	0.1674	0.1833	+0.0159
Accuracy	42.41%	43.95%	+1.54%

**Table 4:** Multi-class classification experiment results (2): 13 classes of abnormal behaviors.

Metric	Original Video	Afterimage-Based Video	Difference
Precision	0.1889	0.2038	+0.0149
Recall	0.1657	0.2103	+0.0446
F1-score	0.1606	0.1902	+0.0296
Accuracy	23.53%	27.78%	+4.25%

**Table 5:** GPT-4 analysis results of afterimages generated from various video scenarios.

Prompt			
This motion history image captures a person’s actions over time. Please analyze the motion trails visible in the image: Identify the key areas where motion is most prominent. Describe the sequence of movements these trails suggest, focusing on how they change and evolve. Based on these motion patterns, interpret what specific action or event might be occurring in this scene. Please provide a concise interpretation, concentrating solely on what the visible motion trails reveal about the person’s actions and the unfolding event.			
(a)	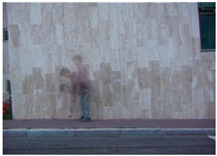		**GPT-4:** The image’s sequence of motion suggests that over some period, **the person stopped at this location, bent down, interacted with the ground in some manner (perhaps touching or picking something up), and then returned to a standing position.** The blurred and overlapping frames indicate a fluid, continuous motion rather than abrupt movements, which might imply a casual or deliberate action.
	**Original Video**	Weizmann datasets denis_bend	**Description of original video**: A scene where a man bends down and then straightens up again on a street.
(b)	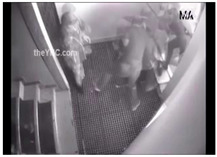		**GPT-4:** The image captures a moment of intense motion involving multiple individuals. The blurred and overlapping figures indicate a chaotic and rapid sequence of actions, where several people appear to be interacting physically. **The motion trails suggest aggressive or forceful movements, as the individuals seem to be pushing or shoving each other within a confined space.**
	**Original Video**	UCF-Crime Assault003_x264	**Description of original video**: A scene showing two men fighting in front of stairs on the right side, while a woman is taking shelter on the left side.
(c)	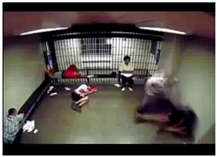		**GPT-4:** The image portrays a scene in a detention or holding cell, where several individuals are present, some sitting or lying down passively, while a significant amount of motion is concentrated on the right side of the frame. **The blurred figures on the right suggest a rapid and forceful altercation between two individuals. The motion blur and overlapping forms indicate vigorous physical action, such as a struggle or fight, characterized by fast-paced movement.**
	**Original Video**	UCF-Crime Fighting011_x264	**Description of original video**: A scene inside a prison where one man is lying collapsed in the center, while on the right side, one man is assaulting another man.

## Data Availability

The UCF-CRIME dataset and Weizmann dataset presented in this study are openly available in the UCF Center for Research in Computer Vision (CRCV) repository at https://www.crcv.ucf.edu/projects/real-world/ and the Weizmann Institute of Science repository at https://www.wisdom.weizmann.ac.il/~vision/SpaceTimeActions.html, respectively (both accessed on 15 October 2024).
